# First record of *Trissolcusbasalis* (Hymenoptera: Scelionidae) parasitizing *Halyomorphahalys* (Hemiptera: Pentatomidae) in the United States

**DOI:** 10.3897/BDJ.7.e39247

**Published:** 2019-10-28

**Authors:** Rammohan R Balusu, Elijah J Talamas, Ted E Cottrell, Michael D Toews, Brett R Blaauw, Ashfaq A Sial, David G Buntin, Henry Y Fadamiro, Glynn Tillman

**Affiliations:** 1 Department of Entomology and Plant Pathology, Auburn University, Auburn, AL, United States of America Department of Entomology and Plant Pathology, Auburn University Auburn, AL United States of America; 2 Florida State Collection of Arthropods, Gainesville, United States of America Florida State Collection of Arthropods Gainesville United States of America; 3 USDA/SEL, Washington, DC, United States of America USDA/SEL Washington, DC United States of America; 4 United States Department of Agriculture, Agricultural Research Service, Southeastern Fruit & Nut Tree Research Laboratory, Byron, GA, United States of America United States Department of Agriculture, Agricultural Research Service, Southeastern Fruit & Nut Tree Research Laboratory Byron, GA United States of America; 5 Department of Entomology, University of Georgia, Tifton, GA, United States of America Department of Entomology, University of Georgia Tifton, GA United States of America; 6 Department of Entomology, University of Georgia, Athens, GA, United States of America Department of Entomology, University of Georgia Athens, GA United States of America; 7 Department of Entomology, University of Georgia, Athens, GA, United States of America Department of Entomology, University of Georgia Athens, GA United States of America; 8 Department of Entomology, University of Georgia, Griffin, GA, United States of America Department of Entomology, University of Georgia Griffin, GA United States of America; 9 United States Department of Agriculture, Agricultural Research Service, Crop Protection & Management Research Laboratory, Tifton, GA, United States of America United States Department of Agriculture, Agricultural Research Service, Crop Protection & Management Research Laboratory Tifton, GA United States of America

**Keywords:** Parasitoid wasp, endoparasitoid, brown marmorated stink bug

## Abstract

**Background:**

A parasitoid wasp, *Trissolcusbasalis* (Wollaston), was recorded parasitizing eggs of the invasive stink bug *Halyomorphahalys* (Stål) in the United States. This is the first record of this species parasitizing fresh and frozen eggs of *H.halys* in the United States.

**New information:**

First record of *Trissolcusbasalis parasitizing Halyomorphahalys* eggs in the United States.

## Introduction

The brown marmorated stink bug, *Halyomorphahalys* (Stål), 1855 (Hemiptera: Pentatomidae) (BMSB) is a native of China, Taiwan, South Korea and Japan. Unfortunately, this invasive insect pest has spread to the United States (Lee et al. 2013), where it is both an urban nuisance pest (Inkley 2012) and a serious economic pest of orchard, field and vegetable crops (Leskey et al. 2012a, Leskey et al. 2012b, Rice et al. 2014). The first known *H.halys* populations in the United States were reported in 1996 from Allentown, PA (Hoebeke and Carter 2003). It has now been found in 44 states (StopBMSB 2018).

In the south-eastern U.S., populations of *H.halys* are continuing to expand into the Piedmont and Coastal Plains regions of Georgia and Alabama. *Halyomorphahalys* was first detected in Alabama in 2010. One year later, urban pest management professionals began reporting overwintering brown marmorated stink bugs in homes in the metropolitan Atlanta area. Currently, the brown marmorated stink bug threatens peaches, plums, blueberries, apples, wine grapes, kiwifruit, soybean, cotton, pecan and tomatoes in both states. The tree of heaven, *Ailanthusaltissima* (Mill.) Swingle, a tree with seed pods that are a favourite non-crop food source for *H.halys*, also occurs in both states.

Presently, 18 species of hymenopteran endoparasitoids in the genera *Anastatus* Motchulsky (Eupelmidae), *Trissolcus* Ashmead, *Telenomus* Haliday and *Gryon* Haliday (Scelionidae) have been reported to parasitize eggs of *H.halys* in the U.S. (Abram et al. 2014, Rice et al. 2014). As the impact of stink bug parasitoids on this pest was unknown in Georgia and Alabama, a survey to examine parasitism and species composition of parasitoids attacking sentinel egg masses of *H.halys* was conducted in 2017 in regions where populations of *H.halys* had become established.

## Materials and methods

Laboratory-reared *H.halys* egg masses were laid on knit cloth (97% cotton, 3% spandex). On 24 June 2018, 30 fresh egg masses (≤ 24 h old) were hung as sentinels on tomato plants for 72 h. Some egg masses (≤ 12 h old) were frozen and held at -20ºC for 1–4 d. On 18 October, 30 frozen egg masses were hung as sentinels on plants in cotton and soybean for 72 h. In the laboratory, the collected egg masses were held for emergence of adult parasitoids and emergent wasps were identified using the key of Talamas et al. (2015). Voucher specimens of parasitoids are deposited in the Florida State Collection of Arthropods, Gainesville, Florida (FSCA 00090444, FSCA 00090269).

All egg masses were dissected for dead, immature parasitoids. Determination of *T.basalis* immature stages, mainly third instars, prepupae and pupae, were based on descriptions of *T.basalis* immatures in Volkoff and Colazza (1992) and on descriptions of *H.halys* eggs, parasitised by *T.basalis* every 24 h from oviposition to pupation (G. Tillman, unpublished data).

Two dried point-mounted specimens were selected for DNA extraction and mitochondrial cytochrome c oxidase I (COI) fragment sequencing. Specimens were softened in 70% ethanol for two hours, then DNA was extracted using a DNeasy Blood and Tissue Kit (Qiagen). The DNA samples were quantified using a NanoDrop 2000 spectrophotometer (Thermo Scientific). At least 20 ng of genomic DNA was used per PCR. The 5’-COI region was PCR-amplified using the primers LCO1490 and HCO2198 (Folmer et al. 1994). PCRs were performed at 25 µl volumes using HiFi HotStart DNA Polymerase (Kapa Biosystems). PCR thermocycle conditions were: 1) initial denaturing at 95°C for 2 min followed by 32 cycles of steps 2–4, 2) 98°C for 30 seconds, 3) 50°C for 30 seconds, 4) 72°C for 40 seconds and 5) final extension at 72°C for 7:00 minutes. PCR products were verified by gel electrophoresis and cleaned for sequencing with QIAquick Gel Extraction Kits (Qiagen). Purified PCR products were Sanger-sequenced in both directions using BigDye Terminator v3.1 (Applied Biosystems) chemistry on a SeqStudio Genetic Analyzer (Applied Biosystems). Sequence reads were trimmed and sequence contigs were assembled in Sequencher 5.4.6 (Gene Codes Corporation). COI sequences, generated during this study, were deposited in GenBank (MK720833, MK720834).

## Taxon treatments

### Trissolcus basalis

(Wollaston) 1858

9B7BFB49-9F19-5847-91C4-1010F575113F

#### Materials

- **Type status:** Other material. **Occurrence:** catalogNumber: FSCA 00090269; recordedBy: Balusu, R. (Rammohan); individualCount: 1; sex: female; lifeStage: adult; occurrenceID: urn:lsid:biosci.ohio-state.edu:osuc_occurrences:FSCA__00090269; **Taxon:** scientificNameID: urn:lsid:biosci.ohio-state.edu:osuc_names:3189; scientificName: Trissolcusbasalis; kingdom: Animalia; phylum: Arthropoda; class: Hexapoda; order: Hymenoptera; family: Scelionidae; genus: Trissolcus; specificEpithet: basalis; **Location:** country: United States; stateProvince: Alabama; county: Tuscaloosa; locality: Tuscaloosa, Tuscaloosa Co., AL, U.S.A.; decimalLatitude: 33.21; decimalLongitude: -87.57; georeferenceSources: GNIS-USGS; **Identification:** identifiedBy: Talamas, E. J. (Elijah Jacob); dateIdentified: 2019; **Event:** samplingProtocol: reared from egg; eventDate: 06/17/2017; verbatimEventDate: Jun-17-2017; fieldNotes: [USA: AL: Tuscaloosa. Tomato 6-2, ex. fresh BMSB eggs 17-JUN-2017, Coll. Rammohan Balusu]; **Record Level:** language: en; institutionCode: Florida State Collection of Arthropods, Gainesville, FL (FSCA); collectionCode: Insects; basisOfRecord: PreservedSpecimen; source: http://hol.osu.edu/spmInfo.html?id=FSCA%2000090269
- **Type status:** Other material. **Occurrence:** catalogNumber: FSCA 00090444; recordedBy: Balusu, R. (Rammohan); individualCount: 1; sex: female; lifeStage: adult; occurrenceID: urn:lsid:biosci.ohio-state.edu:osuc_occurrences:FSCA__00090444; **Taxon:** scientificNameID: urn:lsid:biosci.ohio-state.edu:osuc_names:3189; scientificName: Trissolcusbasalis; kingdom: Animalia; phylum: Arthropoda; class: Hexapoda; order: Hymenoptera; family: Scelionidae; genus: Trissolcus; specificEpithet: basalis; **Location:** country: United States; stateProvince: Alabama; county: Tuscaloosa; locality: Tuscaloosa, Tuscaloosa Co., AL, U.S.A.; decimalLatitude: 33.21; decimalLongitude: -87.57; georeferenceSources: GNIS-USGS; **Identification:** identifiedBy: Talamas, E. J. (Elijah Jacob); dateIdentified: 2019; **Event:** samplingProtocol: reared from egg; eventDate: 07/23/2017; verbatimEventDate: Jul-23-2017; fieldNotes: [USA: AL: Tuscaloosa, Tomato 3-6, ex. fresh BMSB eggs 23-JUL-2017, Coll. Rammohan Balusu]; **Record Level:** language: en; institutionCode: Florida State Collection of Arthropods, Gainesville, FL (FSCA); collectionCode: Insects; basisOfRecord: PreservedSpecimen; source: http://hol.osu.edu/spmInfo.html?id=FSCA%2000090444

#### Diagnosis

**Figure 1.**
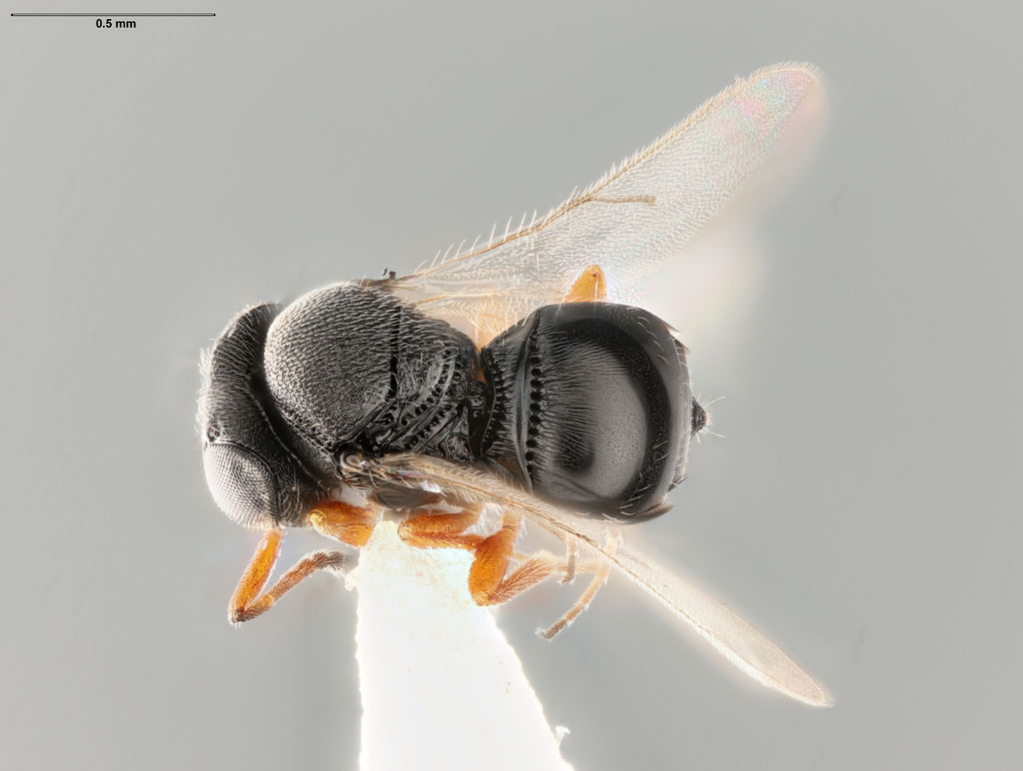
*Trissolcusbasalis*, female (FSCA 00090269), dorsal habitus.

**Figure 2.**
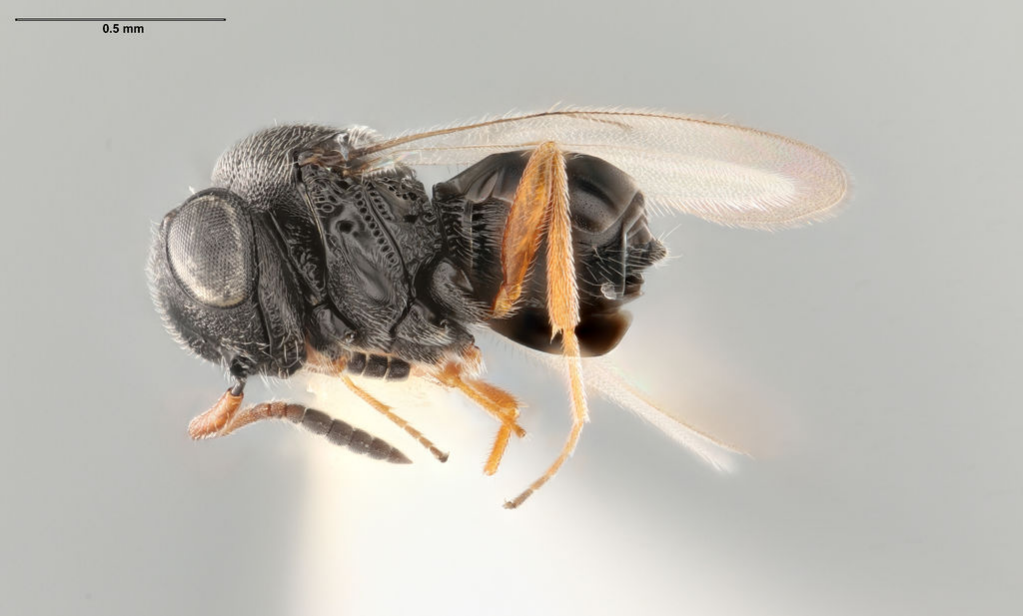
*Trissolcusbasalis*, female (FSCA 00090269), lateral habitus.

**Figure 3.**
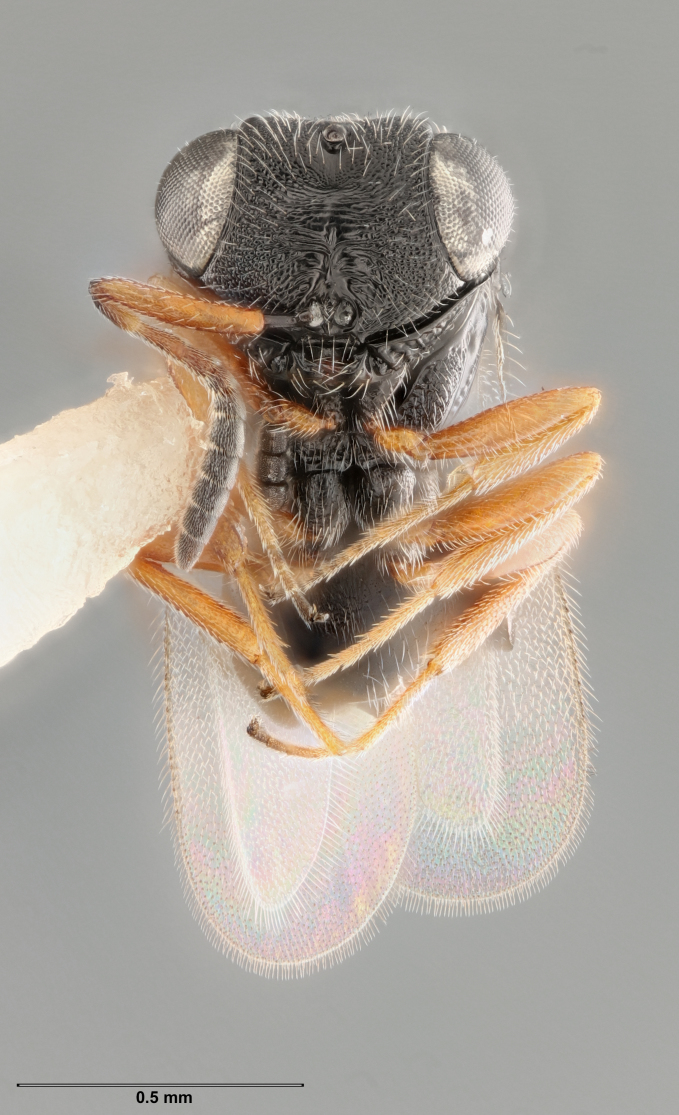
*Trissolcusbasalis*, female (FSCA 00090269), anteroventral habitus.

*Trissolcusbasalis* can be identified from Nearctic congeners by the combination of the following characters: vertex without hyperoccipal carina, netrion sulcus incomplete, mesopleuron with episternal foveae shallowly impressed, metapleuron without setation and without well-defined paracoxal sulcus; T2 striate (Figs 1, 2, 3) (Talamas et al. 2015).

#### Distribution

*Trissolcusbasalis* is found worldwide (http://hol.osu.edu/map-large.html?id=3189).

#### Ecology

At the Tuscaloosa site, *T.basalis* parasitised four of the 30 sentinel egg masses; at the other two sites, only one of the 30 egg masses was parasitised by *T.basalis*. Overall, percent parasitism per egg mass was moderately high (62.7%). In general, percent immature mortality was slightly higher for frozen egg masses (48.2%) than for fresh ones (35.4%). Overall, 38.0% of the parasitoids emerged as adults. A female biased sex ratio of 4F:1M was observed for emergent parasitoids.

#### Biology

Additional host associations of *T.basalis*, provided by Johnson (1985), are *Nezaraviridula* (L.), *Euschistusservus* (Say), *Euthyrhynchusfloridanus* (L.), *Piezodorushybneri* (Gmelin) and *Plautiaaffinis* (Dallas). Additional host associations, provided by Talamas et al. (2015), are *Aeliaacuminata* (L.), *Aeliacognata* Fieber, *Aeliagermari* Küster, *Agonoscelisrutila* (Fabricius), *Calideadregeii* Germar, *Carpocorisfuscispinus* (Boheman), *Coleotichus blackburniae, Cuspicona simplex* Walker, *Dolicorisbaccharum* (L.), *Eurydemaornata* (L.), *Eurygasteraustriaca* (Schrank), *Eurygasterintegriceps* Puton, *Graphosomasemipunctata* (Fabricius), *Halyomorphaannulicornis* (Signoret), *Odontotarsusgrammicus* (L.), *Oechaliaschellenbergi* Guérin-Méneville and *Raphigaster* Laporte.

#### Taxon discussion

The CO1 sequences of the two specimens were identical to each other and to the 7 CO1 sequences of *T.basalis* in Genbank. These sequences derive from specimens collected in Italy, Japan and the United States and their invariance indicates that this gene is not informative for identifying populations within the species.

## Discussion

Multiple species of *Trissolcus* are known to oviposit into the eggs of *H.halys* despite a physiological inability to develop in them, creating an evolutionary trap (Abram et al. 2014, Haye et al. 2015, Abram et al. 2017). Our records of *T.basalis* are mainly from fresh BMSB eggs, providing evidence that *T.basalis* finds live BMSB eggs acceptable and suitable as a host. *Trissolcusbasalis* belongs to the *basalis*-species group (sensu Johnson 1985, Talamas et al. 2017), whereas the species reported to successfully parasitise BMSB in its native range belong to the *flavipes*-group (sensu Talamas et al. 2017). Our records demonstrate that species in the *basalis* group also can successfully parasitise BMSB. In the Umbria Region of Italy, *T.basalis* emerged from frozen eggs of *H.halys* in soybean (Rondoni et al. 2017). This parasitoid was also reported parasitising *H.halys* in Lengquan, China, at very low frequency (Zhang et al. 2017). Peach leaves with naturally-laid egg masses, either fresh or stored at 10°C for no more than 48 h to prevent further development, were deployed as sentinels in the field. At the temperature used, the stored eggs were likely alive when deployed, for BMSB egg masses need to be held in a refrigerator at a minimum of 3.9°C for 12 h to kill the eggs (G. Tillman, unpublished data). At present, reports of parasitism of BMSB by *T.basalis* are rare.

## Supplementary Material

XML Treatment for Trissolcus basalis

## References

[B5234575] Abram K., Gariepy D., Boivin G., Brodeur J. (2014). An invasive stink bug as an evolutionary trap for an indigenous egg parasitoid. Biological Invasions.

[B5234532] Abram P., Hoelmer K. A., Acebes-Doria A. L., Andrews H., Beers E. H., Bergh J. C. (2017). Indigenous arthropod natural enemies of the invasive brown marmorated stink bug in North America and Europe. Journal of Pest Science.

[B5234652] Folmer O., Black M., Hoeh W., Lutz R., Vrijenhoek R. (1994). DNA primers for amplification of mitochondrial cytochrome c oxidase subunit I from diverse metazoan invertebrates. Molecular Marine Biology and Biotechnology.

[B5234663] Haye T., Fischer S., Zhang J., Gariepy T. (2015). Can native egg parasitoids adopt the invasive brown marmorated stink bug, *Halyomorphahalys* (Heteroptera: Pentatomidae), in Europe?. Journal of Pest Science.

[B5234714] Hoebeke R., Carter E. (2003). *Halyomorphahalys* (Stål) (Heteroptera: Pentatomidae): a polyphagous plant pest from Asia newly detected in North America. Proceedings of the Entomological Society of Washington.

[B5234724] Inkley B. (2012). Characteristics of home invasion of the brown marmorated stink bug (Hemiptera: Pentatomidae). Journal of Entomological Science.

[B5234734] Johnson F. (1985). Systematics of New World *Trissolcus* (Hymenoptera: Scelionidae): species related to *T.basalis*. The Canadian Entomologist.

[B5234880] Lee Doo-Hyung, Short Brent D., Joseph Shimat V., Bergh J. Christopher, Leskey Tracy C. (2013). Review of the biology, ecology, and management of *Halyomorphahalys* (Hemiptera: Pentatomidae) in China, Japan, and the Republic of Korea. Environmental Entomology.

[B5234768] Leskey T., Short B., Butler B., Wright S. (2012). Impact of the invasive brown stink bug, *Halyomorphahaly*s (Stål), in mid-Atlantic fruit orchards in the United States: Case studies of commercial management. Psyche: A Journal of Entomology.

[B5234852] Leskey Tracy C., Hamilton George C., Nielsen Anne L., Polk Dean F., Rodriguez-Saona Cesar, Bergh J. Christopher, Herbert D. Ames, Kuhar Tom P., Pfeiffer Douglas, Dively Galen P., Hooks Cerruti R. R., Raupp Michael J., Shrewsbury Paula M., Krawczyk Greg, Shearer Peter W., Whalen Joanne, Koplinka-Loehr Carrie, Myers Elizabeth, Inkley Douglas, Hoelmer Kim A., Lee Doo-Hyung, Wright Starker E. (2012). Pest status of the brown marmorated stink Bug, *Halyomorphahalys* in the USA. Outlooks on Pest Management.

[B5234817] Rice Kevin B., Bergh Chris J., Bergmann Erik J., Biddinger Dave J., Dieckhoff Christine, Dively Galen, Fraser Hannah, Gariepy Tara, Hamilton George, Haye Tim, Herbert Ames, Hoelmer Kim, Hooks Cerruti R., Jones Ashley, Krawczyk Greg, Kuhar Thomas, Martinson Holly, Mitchell William, Nielsen Anne L., Pfeiffer Doug G., Raupp Michael J., Rodriguez-Saona Cesar, Shearer Peter, Shrewsbury Paula, Venugopal P. Dilip, Whalen Joanne, Wiman Nik G., Leskey Tracy C., Tooker John F. (2014). Biology, ecology, and management of brown marmorated stink bug (Hemiptera: Pentatomidae). Journal of Integrated Pest Management.

[B5234921] Rondoni Gabriele, Bertoldi Valeria, Malek Robert, Foti Maria Cristina, Peri Ezio, Maistrello Lara, Haye Tim, Conti Eric (2017). Native egg parasitoids recorded from the invasive *Halyomorphahalys* success fully exploit volatiles emitted by the plant–herbivore complex. Journal of Pest Science.

[B5234935] StopBMSB Biology, ecology, and management of the brown marmorated stink bug in specialty crops. http://www.stopbmsb.org.

[B5234954] Talamas Elijah J., Johnson Norman F., Buffington Matthew (2015). Key to Nearctic species of *Trissolcus* Ashmead (Hymenoptera, Scelionidae), natural enemies of native and invasive stink bugs (Hemiptera, Pentatomidae). Journal of Hymenoptera Research.

[B5234944] Talamas Elijah J., Buffington Matthew L., Hoelmer Kim (2017). Revision of Palearctic *Trissolcus* Ashmead (Hymenoptera, Scelionidae). Journal of Hymenoptera Research.

[B5234986] Volkoff Nathalie, Colazza Stefano (1992). Growth patterns of teratocytes in the immature stages of *Trissolcusbasalis* (Woll.) (Hymenoptera: Scelionidae), an egg parasitoid of *Nezaraviridula* (L.) (Heteroptera : Pentatomidae). International Journal of Insect Morphology and Embryology.

[B5235006] Zhang Jinping, Zhang Feng, Gariepy Tara, Mason Peter, Gillespie Dave, Talamas Elijah, Haye Tim (2017). Seasonal parasitism and host specificity of *Trissolcusjaponicus* in northern China. Journal of Pest Science.

